# Aggregation-Induced Emission of Au/Ag Alloy Nanoclusters for Fluorescence Detection of Inorganic Pyrophosphate and Pyrophosphatase Activity

**DOI:** 10.3389/fbioe.2020.628181

**Published:** 2021-01-15

**Authors:** Zhongli Lei, Jie Zhou, Miao Liang, Yan Xiao, Zhihong Liu

**Affiliations:** Hubei Collaborative Innovation Center for Advanced Organic Chemical Materials, Ministry of Education Key Laboratory for the Synthesis and Application of Organic Functional Molecules & College of Chemistry and Chemical Engineering, Hubei University, Wuhan, China

**Keywords:** Au/Ag alloy nanoclusters, aggregation-induced emission, fluorescence detection, pyrophosphate, pyrophosphatase

## Abstract

The development of sensitive and accurate detection of inorganic pyrophosphate (PPi) and pyrophosphatase activity (PPase) is important as they play vital roles in biological systems. However, it is still not satisfactory for most of the analytical methods for PPi and PPase because of their Cu^2+^-dependence and poor accuracy. Although the metal ion triggered aggregation-induced emission (AIE) of metal nanoclusters (NCs) offers a new approach to design a Cu^2+^-free strategy for the accurate determination of PPi and PPase recently, current methods are all focused on utilizing pure metal NCs. Alloy NCs incorporating the advantages of diverse metal usually can achieve improved behaviors in the application, such as enhanced sensitivity and stability. In this work, glutathione stabilized alloy Au/Ag NCs were synthesized via a simple method and used for the fluorescence detection of PPi and PPase based on a Zn^2+^-regulated AIE strategy. The controlled release of Zn^2+^ by PPi and PPase could regulate the AIE of Au/Ag NCs and be employed to response PPi concentration and PPase activity. This method processes simple procedure, high sensitivity and stability, and low toxicity. In addition, we also studied the AIE behaviors of this Au/Ag NCs and offer some fundamental understanding of the AIE properties of water-soluble alloy NCs. This study not only provides a straightforward and new approach for PPi and PPase determination but a basis for further study on the AIE properties of alloy NCs and their application.

## Introduction

Inorganic pyrophosphatase (PPase) is a kind of acid anhydride hydrolysis enzyme that works on a double phosphate bond. It can catalyze the hydrolysis of inorganic pyrophosphate (PPi) to phosphate ions, thereby being responsible for the control of the level of pyrophosphate in biological system (Farquharson, 2018). The PPi and PPase are associated with many important physiological processes, such as lipid metabolism (including lipid synthesis and breakdown), calcium absorption, bone formation, DNA synthesis and other biochemical transformations (Curbo et al., 2006; Ko et al., 2007; Polewski et al., 2010). Hence, the development of sensitive, reliable, and biocompatible PPi and PPase assay methods is of significant importance for the understanding of their biological function and the diagnosis and therapy of many relevant diseases.

To date, several methods including electrochemical (Lin et al., 2016), bioluminescence (Eriksson et al., 2001; Tao et al., 2015), colorimetric (Deng et al., 2013; Shang et al., 2019), and fluorescence (Deng et al., 2016; Sun et al., 2016; Farzin et al., 2020) methods have been developed for the determination of PPi and PPase activity. Among these methods, fluorescent assays show some obvious advantages such as excellent sensitivity and selectivity, flexible visual analysis, fast response, low cost, simple operation, and so on. However, most of the previous fluorescence assays for PPi and PPase were on basis of the high affinity of PPi for Cu^2+^ and the catalytic hydrolysis of PPi by PPase (Xu et al., 2015; Zhou et al., 2016; Zhu et al., 2016). Although these detection methods have made contributions for detecting PPi and PPase, the introduction of Cu^2+^ was likely to have severe toxicity in the biological system and the interference by thiol-containing substances in the surrounding biological environment may be significant. Moreover, Cu^2+^ served as a quencher for the fluorescent materials in these assays and thus these Cu^2+^-associated assays may be easily disturbed by other quenchers. Therefore, it is still highly desirable to developing reliable assays for PPi and PPase that does not involving Cu^2+^.

Metal nanoclusters (M NCs), as a type of promising luminescent material, are widely used in bioanalysis owing to their ultra-small size, simple synthesis method, tunable emission, large stokes shift and high biocompatibility (Tao et al., 2015; Shang et al., 2019; Farzin et al., 2020) In recent years, some Cu^2+^-free fluorescence analysis approaches for PPi and PPase have been developed by virtue of the attractive aggregation-induced emission (AIE) properties of M NCs, opening a new door for the design of detection systems for PPi and PPase. For example, Tang et al. reported the detection of PPase activity employing Al^3+^ triggered AIE of Ag NCs (Tang et al., 2017). The Ag NCs could combine with Al^3+^ and further form aggregates, resulting in the suppressions of the vibration and rotation of ligands of Ag NCs and non-radiative energy relaxation of excited Ag NCs. Thereby, the emission of Ag NCs could be largely promoted upon aggregating. PPi, which is a hydrolytic substrate of PPase, could remove Al^3+^ from the aggregates by the strong coordination between Al^3+^ and PPi. The controlled release of Al^3+^ by PPase could be employed to response PPase activity. Similarly, Zhu and co-workers established a detection method based on Al^3+^ triggered AIE of Cu NCs (Ye et al., 2019). Zhao, et al. proposed the detection of PPi and PPase using Zn^2+^-triggered AIE of dual ligand co-stabilized Au NCs (Zhao et al., 2019). Nevertheless, these reports are all focused on the utilization of the AIE of pure metal NCs. Ag NCs and Cu NCs enjoy the advantages of low cost and abundant resource (Wang et al., 2014; Zheng et al., 2014) while Au NCs have relatively high stability (Xu et al., 2017). Each kind of M NCs has its strengths. Under these circumstances, alloy NCs based on the integration of multiple metals into NCs become attractive as they may incorporate the advantages of different metal NCs and achieve improved behaviors in the application (Zhou et al., 2016; Jia et al., 2020). However, the synthesis and application toward alloy NCs are still in the early stage. To the best of our knowledge, the study of the AIE behaviors of water-soluble alloy NCs and their biosensing applications have not yet been reported.

**Figure 1:**
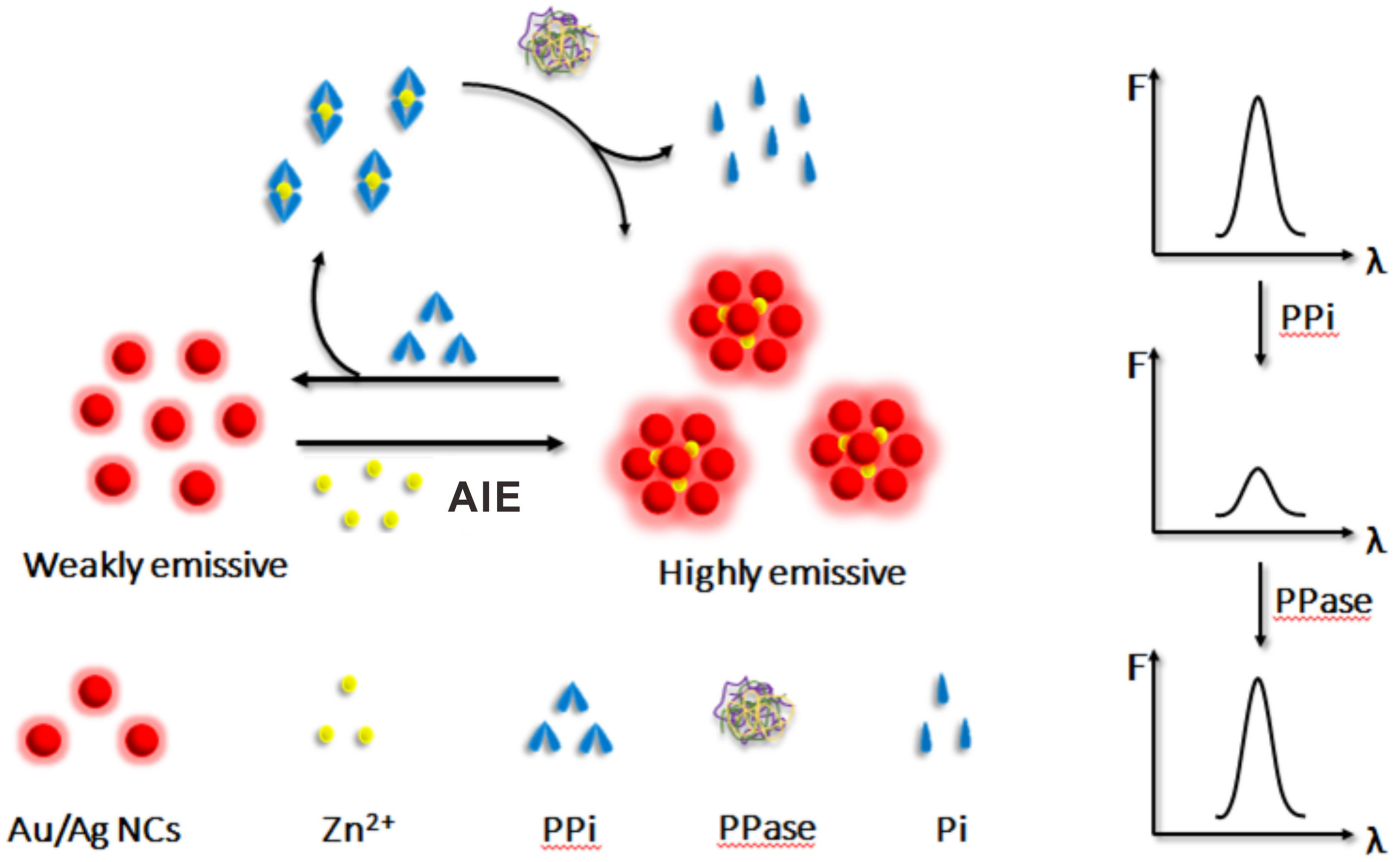
Schematic illustration of the principle of the Zn^2+^-regulated AIE of Au/Ag NCs for inorganic pyrophosphate and pyrophosphatase activity detection.

In this work, we successfully synthesized glutathione stabilized Au/Ag alloy nanoclusters (Au/Ag NCs) via a facile approach and studied the AIE behaviors of these water-soluble Au/Ag NCs. It was found that the as-synthesized alloy NCs exhibited unique AIE characteristics, which were some different from pure metal NCs. We then used the AIE of Au/Ag NCs to construct a simple method for sensitive and reliable determination of PPi and PPase (Figure 1). In our study, Zn^2+^ could effectively cause the aggregation of Au/Ag NCs via the electrostatic interaction between Zn^2+^ and ligands on the surface of Au/Ag NCs, thereby enhancing the luminescence of Au/Ag NCs. When PPi was added to the Zn^2+^-Au/Ag NCs aggregates, the coordination of Zn^2+^ and PPi would remove Zn^2+^ from aggregates (Dong et al., 2017; Xu et al., 2020), resulting in the destruction of aggregates and the decrease of luminescence intensity of Au/Ag NCs. According to the relationship between the fluorescence intensity and the concentration of PPi, the detection of PPi was achieved. When PPase was further added to the above Zn^2+^-Au/Ag NCs-PPi mixing system, it would rapidly decompose of PPi-Zn^2+^ complexes into free Zn^2+^ and phosphate ions. Then the free Zn^2+^ could recombine with Au/Ag NCs, triggering the aggregation of Au/Ag NCs again and the emission enhancement of Au/Ag NCs for the detection of PPase. Benefiting from the employing of Zn^2+^ triggered AIE of Au/Ag alloy NCs, this detection method simultaneously processes high sensitivity and stability, low cytotoxicity as well as simple and convenient operation. This detection mode provides satisfactory results in human serum samples. Besides, this work may offer some basis for understanding the AIE properties of alloy NCs, and also expand its application in biological analysis.

## Experiment

### Materials

Gold (III) chloride trihydrate (HAuCl_4_·3H_2_O), silver nitrate (AgNO_3_), L-glutathione reduced (GSH) pyrophosphatase (PPase) and exonuclease were purchased from Sigma-Aldrich (St. Louis, MO, USA). Streptavidin (SA), bovine serum albumin (BSA), and glucose oxidase (Gox) were obtained from Aladdin Industrial Corporation (Shanghai, China). The rest of the chemical reagents were purchased from Sinopharm Chemical Reagent Co., Ltd. (Shanghai, China) and at least analytical grade. All of the chemicals were used as received without further purification. Ultrapure water (18.25 MΩ·cm) from a Millipore system was used in all aqueous solutions.

### Characterization

The UV-vis absorption spectra were measured on a UV-2550 UV-vis spectrometer (Shimadzu, Japan). The fluorescence measurements were recorded using an RF-5301 PC spectrometer (Shimadzu, Japan). The sizes and morphologies of NCs were characterized by a JEM-2010 transmission electron microscope (TEM) operated at an acceleration voltage of 200 kV. X-ray photoelectron spectroscopy (XPS) spectra were obtained from a Kratos Axis Ultra LD spectrometer using a monochromatic Al Kαsource.

### Synthesis of Au/Ag NCs

The Au/Ag NCs were synthesized by the following simple and convenient method. Briefly, freshly prepared aqueous solutions of GSH (300 μL, 100 mM), AgNO_3_ (125 μL, 20 mM) and HAuCl_4_ solution (375 μL, 20 mM) were added into PBS buffer solution (3.2 mL, 100 mM, pH 8.0) under vigorous stirring. The mixing solution was then heated to 80°C and kept for 3 h. During the heating, the color of the solution changed from colorless to light yellow. Then, the resulting solution was further incubated in the refrigerator (4°C) for about 12 h. Finally, Au/Ag NCs were purified by using a dialysis bag with a molecular weight cutoff (MWCO) of 3,500 Da. After that, orangey-red-emitting Au/Ag NCs were obtained and stored in the refrigerator for further use.

### Aggregation-Induced Emission of Au/Ag NCs

In PBS buffer solution (100 mM, pH 7.0), different concentrations of Zn^2+^ were individually added into Au/Ag NCs solution (0.3 mg/mL) and incubated for 15 min at 37°C. Then, the fluorescence measurements were carried out.

### Determination of PPi in Buffer Solution and Human Serum

Under PBS buffer solution (100 mM, pH 7.0), Au/Ag NCs solution (0.3 mg/mL) was firstly mixed with 400 μM Zn^2+^ and incubated for 15 min at 37°C. Then, different concentrations of PPi were added to the mixed solution and incubated for 5 min at 37°C. Then, the solutions were subjected to fluorescence measurements.

Human serum was diluted 100-fold with 100 mM PBS (pH 7.0) and then added into a centrifugal filtration device (MWCO = 50,000 Da, Millipore) and centrifuged at 6,000 rpm for 20 min. Different concentrations of PPi were added to pretreated human serum samples and incubated with mixed solutions containing Au/Ag NCs (0.3 mg/mL) and Zn^2+^ (400 μM). After reacting at 37°C for 5 min, the fluorescence measurements of mixture solutions were performed.

### Determination of PPase in Buffer Solution and Human Serum

Firstly, 0.3 mg/mL Au/Ag NCs and 400 μM Zn^2+^ were mixed in PBS buffer solution (100 mM, pH 7.0) and incubated for 15 min at 37°C. Then, 400 μM PPi was added to the mixed solution and incubated for another 5 min. Next, different concentrations of PPase were added to the mixed solution. After incubating the mixed solution at 37°C for 1 h, the fluorescence intensity of the analysis system was recorded.

In human serum, standard addition experiments were performed. Different concentrations of PPase were added to pretreated human serum and incubated with a mixed solution containing 0.3 mg/mL Au/Ag NCs, 400 μM Zn^2+^, and 400 μM PPi at pH 7.0. After incubating the mixture at 37°C for 1 h, the fluorescence intensity of Au/Ag NCs was measured.

## Results and Discussion

### Synthesis and Characterization of Au/Ag NCs

**Figure 2:**
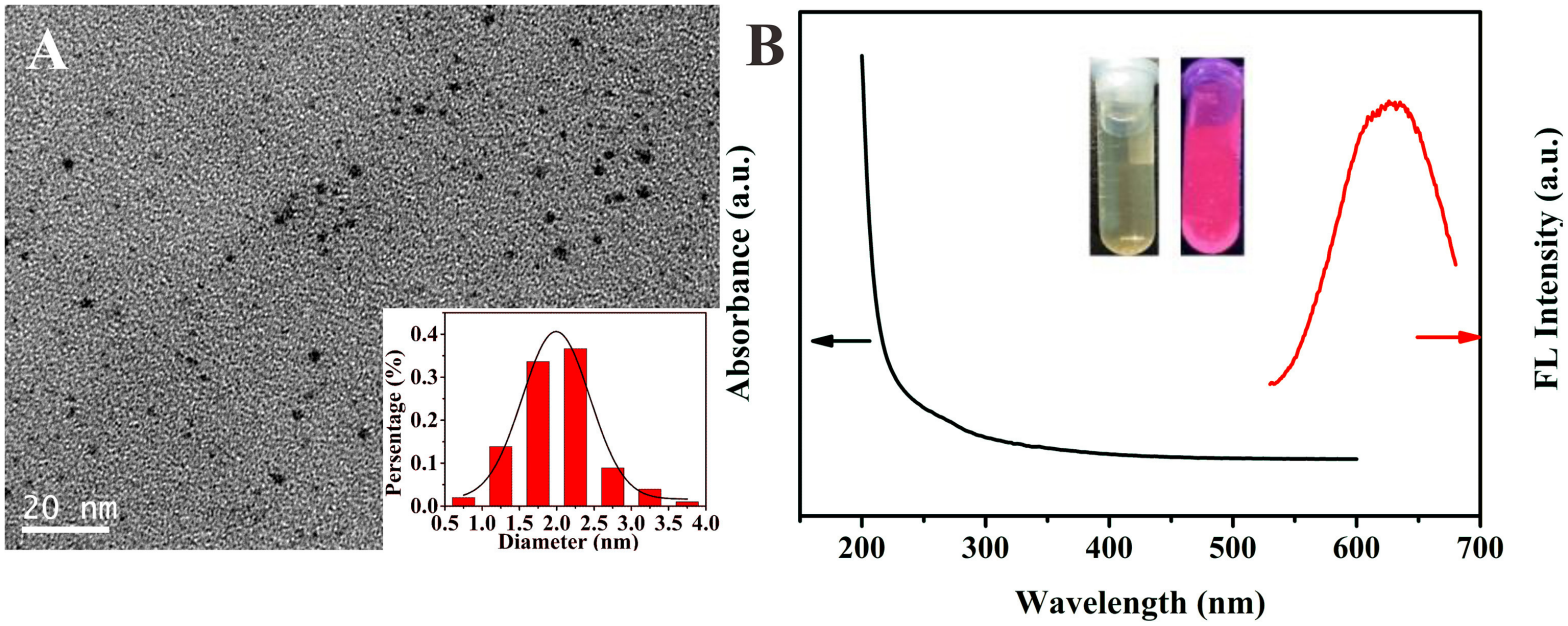
**(A)** Transmission electron microscope (TEM) image of as-prepared Au/Ag NCs and the size distribution of Au/Ag NCs (insert). **(B)** UV-vis absorption spectrum (black line) and fluorescence spectrum (red line) of Au/Ag NCs. Inset is the photograph of Au/Ag NCs solution under natural light (left) and UV irradiation (right).

By simply reducing the mixed metal precursors under the protection of organic ligands, we synthesized water-soluble glutathione-stabilized Au/Ag alloy nanoclusters. The as-synthesized Au/Ag NCs were well-characterized by transmission electron microscope (TEM), X-ray photoelectron spectroscopy (XPS), UV-vis absorption, and fluorescence spectroscopy. The TEM image showed the Au/Ag NCs had a good dispersibility and the average size of the Au/Ag NCs was ~2.0 ± 0.5 nm (Figure 2A and insert). Using XPS analysis, we investigated the elemental composition and oxidation state of Au/Ag NCs. The measurement results showed that Au/Ag NCs were mainly containing the elements of carbon, nitrogen, oxygen, sulfur, gold, and silver, which were corresponding to the raw materials for synthesizing Au/Ag NCs (Supplementary Figure 1A). The high-resolution XPS spectrum for Ag species displayed two different peaks at 367.7 and 373.7 eV, which were attributed to the binding energy of Ag 3d_5/2_ and Ag 3d_3/2_, respectively (Supplementary Figure 1B). The Ag 3d_5/2_ peak were centered at 367.7 eV, between Ag^+^ (367.5 eV) and pure Ag^0^ (368.2 eV), confirming the containing of Ag^0^ in the Au/Ag NCs. As showed in Supplementary Figure 1C, the binding energy of Au 4f_7/2_ and Au 4f_5/2_ were at 84.1 and 87.7 eV, respectively. The Au 4f_7/2_ peak located at 84.1 eV was intermediate between Au(I)-thiolate (86.0 eV) and bulk Au (83.8 eV), suggesting the coexistence of Au^0^ and Au^+^ in the alloy NCs. (Shang et al., 2011; Zhang et al., 2015) These results demonstrated the successful synthesis of alloy Au/Ag NCs. In the FT-IR spectrum of Au/Ag NCs, the absorption peak at 3,420 cm^−1^ was ascribed to the –OH stretching vibration and the peaks at 1,630 and 1,403 cm^−1^ were attributed to the antisymmetric and symmetric stretching vibrations of C=O, respectively (Supplementary Figure 2). These FT-IR data suggested the presence of –COOH on the surface of Au/Ag NCs. Compared with FT-IR spectrum of GSH, the disappearance of the S-H stretching peak at 2,525 cm^−1^ were observed in Au/Ag NCs spectrum indicated the formation of Ag-S or Au-S bonding on the surface of Au/Ag NCs. Then, we studied the optical properties of Au/Ag NCs by UV-vis absorption and fluorescence spectroscopy. As shown in Figure 2B, the as-synthesized Au/Ag NCs had a wide absorption around the UV region and a fluorescence emission peak at 623 nm. Under ultraviolet light irradiation, the naked eye could observe that the Au/Ag NCs aqueous solution showed red luminescence (Figure 2B, Insert).

### Aggregation-Induced Emission of Au/Ag NCs

**Figure 3:**
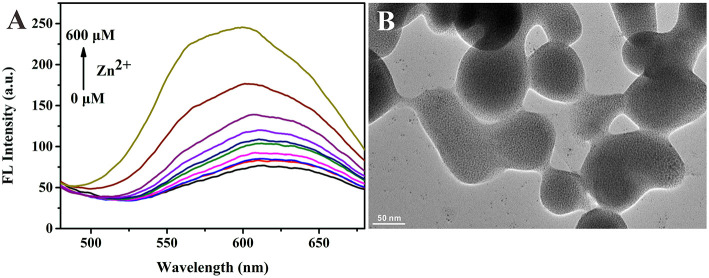
**(A)** Fluorescence spectra of Au/Ag NCs in the presence of different concentrations of Zn^2+^ (0–600 μM). Excitation wavelength: 360 nm. **(B)** Transmission electron microscope (TEM) image of Zn^2+^-Au/Ag NCs aggregates.

Based on the successful preparation of alloy NCs, we next studied the AIE properties of Au/Ag NCs. Because the surface of GSH-capped NCs is negatively charged, the Au/Ag NCs with GSH as ligands could combine with metal cations via electrostatic interaction (Yao et al., 2014). After adding zinc ions into the Au/Ag NCs solution, we observed the luminescence change of Au/Ag NCs. With the increase of their incubation time, the fluorescence intensity of Au/Ag NCs gradually increased and reached a plateau when the incubation time was 15 min (Supplementary Figure 3). In addition, the emission enhancement of Au/Ag NCs highly depended on the concentration of Zn^2+^. As presented in Figure 3A, the fluorescence intensity of Au/Ag NCs was increased with increasing amounts of Zn^2+^. From the TEM image of Zn^2+^-Au/Ag NCs complexes, we could clearly see a lot of aggregates of Au/Ag NCs (Figure 3B). The zeta potential of Au/Ag NCs was changed from −40.3 to −22.9 mV after the addition of Zn^2+^, revealing the occurrence of charge neutralization (Supplementary Figure 4). These results confirmed the Zn^2+^ triggered AIE phenomenon of Au/Ag NCs. It needs to be especially noted that along with the increasing degree of aggregation, the emission of alloy NCs exhibited a blue-shift, which has not occurred in the aggregation of pure metal NCs. This phenomenon indicated that aggregation affected the radiation relaxation process of excited alloy NCs. It is widely recognized that the luminescence of thiolated NCs is originated from ligand-to-metal-metal or ligand-to-metal charge transfer (LMMCT/LMCT) (Li et al., 2019; Yang et al., 2020). Thus, the electronic transition and electronic structure of metal NCs are important factors for the luminescence of NCs. As reported by many previous literatures, the synergistic effect between Au atoms and Ag atoms in the Au/Ag alloy NCs would affect the electronic structure of NCs and thereby the emission wavelength of Au/Ag NCs (Negishi et al., 2010; Ganguly et al., 2016; Soldan et al., 2016; Theivendran et al., 2018). The blue-shift of emission of Au/Ag NCs after aggregating illustrated that the aggregation may not only influence the vibration and rotation of ligand molecules but also the synergistic interaction between different metal atoms, further changing the electron relaxation process in alloy NCs. Our results indicated the AIE behaviors of alloy NCs are more complicated than that of pure metal NCs. More efforts need to be devoted to the detailed mechanism of the AIE properties of alloy NCs in the future.

### The Principle of the PPi and PPase Detection

After confirming the AIE phenomenon of Au/Ag NCs, we constructed a simple strategy for the detection of PPi and PPase based on the Zn^2+^-regulated AIE of Au/Ag alloy NCs (Figure 1). Upon the presence of Zn^2+^, the aggregation of Au/Ag NCs would occur via the electrostatic interaction between Zn^2+^ and Au/Ag NCs. Due to the restrain of the intramolecular vibration and rotation of ligands by the aggregation, the emission of Au/Ag NCs could be enhanced. PPi, containing two phosphate groups, could coordinate well with Zn^2+^ (Xu et al., 2020). Therefore, when PPi were present, they could competitively bind with Zn^2+^ and remove Zn^2+^ from aggregates. The destruction of the aggregation effect would reduce the luminescence of Au/Ag NCs that could be used to determine the concentration of PPi. PPase can effectively catalyze the hydrolysis of PPi to single phosphate group and hence breakdown the as-formed PPi-Zn^2+^ complexes. The release of Zn^2+^ from the PPi-Zn^2+^ complexes could re-form the aggregation of Au/Ag NCs and enhance their emission again. A relationship between the recovery of fluorescence intensity and the amount of PPase could be established for PPase activity analysis.

### Fluorescence Determination of PPi

**Figure 4:**
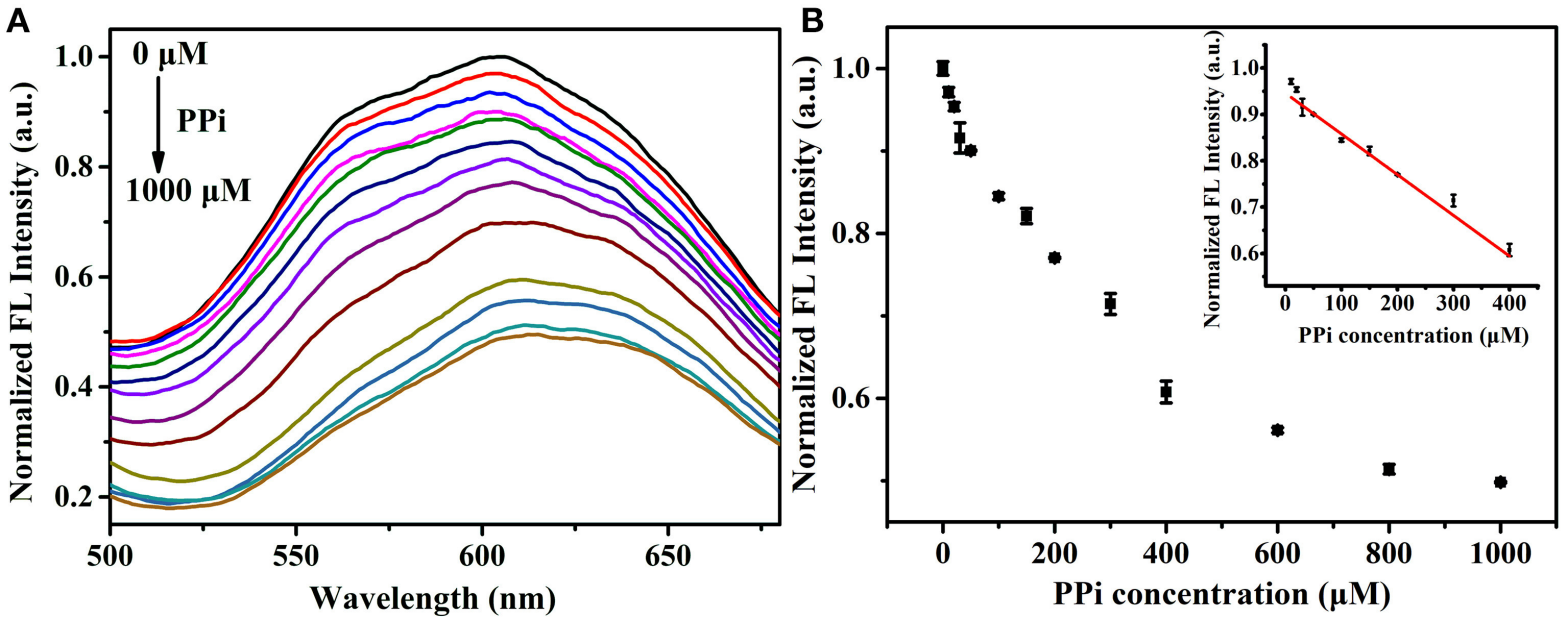
**(A)** Fluorescence spectra of Zn^2+^-Au/Ag NCs aggregates in the presence of different concentration of PPi (0–1,000 μM). Excitation wavelength: 360 nm **(B)** The fluorescence intensity of Au/Ag NCs in response to different concentration of PPi (0–1,000 μM). Inset: the linear relationship between the fluorescence intensity of NCs and PPi concentration.

We first examined the response of the proposed analysis method to PPi. When adding PPi into the Zn^2+^-Au/Ag NCs aggregates, the strong coordination between PPi and Zn^2+^ would breakdown the aggregation of Au/Ag NCs, which accompanies the reduction of the fluorescence of Au/Ag NCs. The experimental results were in line with our expectations. Upon the introduction of PPi into the Zn^2+^-Au/Ag NCs aggregates solution, the fluorescence intensity of the system decreased rapidly and almost in 2 min a quenching plateau could be observed (Supplementary Figure 5). The fast response is beneficial to the target analysis because of its time-saving. Furthermore, the dynamic light scattering (DLS) analysis presented the size of Au/Ag NCs-Zn^2+^ aggregate was decreased from 340 ± 20 nm to 205 ± 6 nm after the addition of PPi (Supplementary Figure 6), indicating the breakdown of Au/Ag NCs-Zn^2+^ aggregates by PPi. To confirm our detection principle and exclude the effect of PPi per se on the emission of Au/Ag NCs, we examined the emission change of Au/Ag NCs after the introduction of PPi. The result showed the PPi did not affect the emission of Au/Ag NCs (Supplementary Figure 7). To evaluate the analytical performance of this method for PPi, the fluorescence spectra of the Zn^2+^-Au/Ag NCs aggregates solution incubating with different concentrations of PPi were recorded. The emission intensity of Au/Ag NCs was decreased along with the increase of the concentrations of PPi (Figures 4A,B). A good linear relationship between the fluorescence intensity of Au/Ag NCs and PPi concentrations over the range of 10–400 μM was obtained from our analysis strategy (Figure 4B, insert). The linear regression equation is y = −0.1512 x + 163.72 (R^2^ = 0.9939), where y and x are the fluorescence intensity of Au/Ag NCs and PPi concentration, respectively. When the signal-to-noise ratio is 3.0, the detection limit (LOD) was calculated to be 3.2 μM.

The high selectivity is also an important issue for a good analysis system. In order to assess the selectivity of Zn^2+^-Au/Ag NCs aggregates for PPi detection, we investigated the response of the Zn^2+^-Au/Ag NCs aggregates toward different anions, including F^−^, Cl^−^, Br^−^, I^−^, ${\text{SO}}_{4}^{2-}$, ${\text{NO}}_{3}^{-}$, Ac^−^, ${\text{CO}}_{3}^{2-}$, ${\text{HCO}}_{3}^{-}$, ${\text{PO}}_{4}^{3-}$, ${\text{HPO}}_{4}^{2-}$, and H_2_${\text{PO}}_{4}^{-}$ (Supplementary Figure 8). The experimental results showed that only PPi could quench the emission of Zn^2+^-Au/Ag NCs aggregates distinctly, indicating that this method has good selectivity for PPi.

Using the standard addition method, we further verified the utility of the proposed detection method in human serum samples. To test the sample recovery of this analysis method, four concentrations of PPi (50.0, 150.0, 200.0, and 300.0 μM) were spiked into 1% diluted human serum and the detection was carried out under the same condition with the analysis in buffer. As displayed in Supplementary Table 1, the obtained recoveries ranged from 94.0 to 102.7%, and RSD was between 0.6 and 2.5%. These results strongly demonstrated the reliability and suitability of the proposed approach for practical applications.

### Fluorescence Determination of PPase

**Figure 5:**
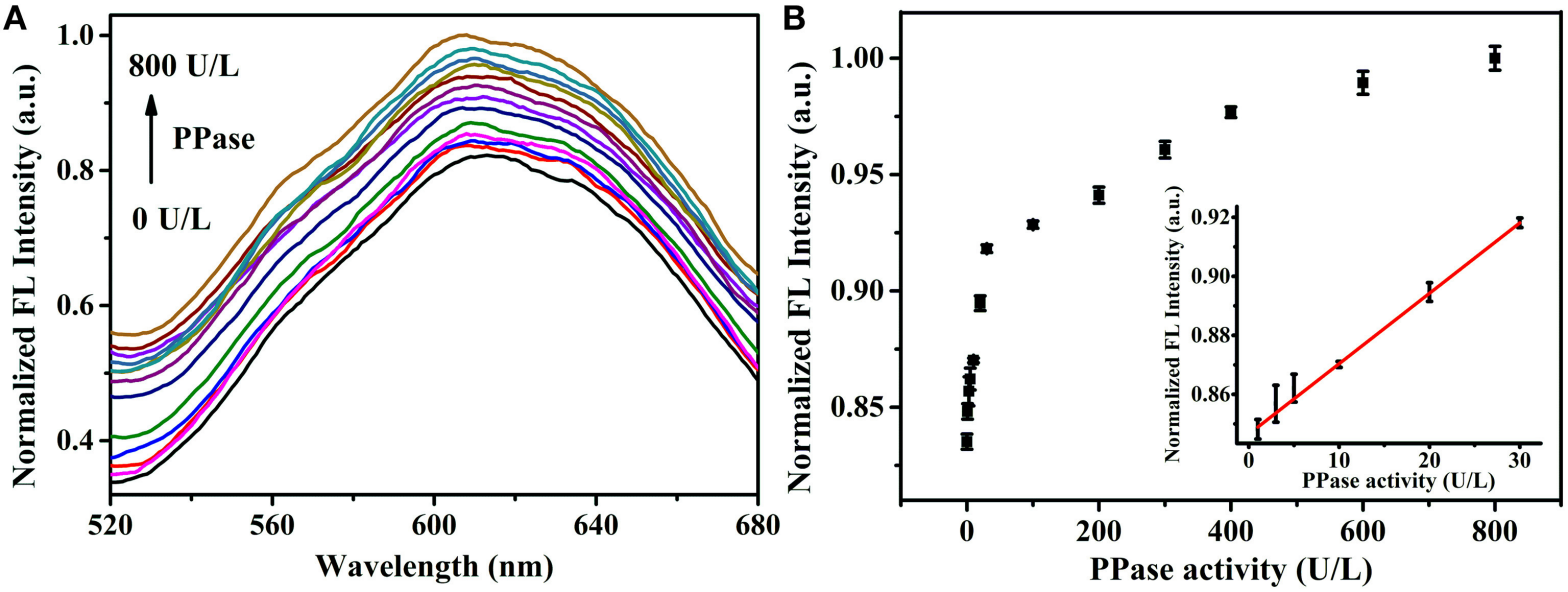
**(A)** Fluorescence spectra of Zn^2+^-Au/Ag NCs-PPi analysis system in the presence of different concentrations of PPase (0–800 U/L). Excitation wavelength: 360 nm **(B)** The fluorescence intensity of Zn^2+^-Au/Ag NCs-PPi analysis system in response to different concentrations of PPase (0–800 U/L). Inset: the linear relationship between the fluorescence intensity of NCs and PPase concentration.

Since the PPi could be hydrolyzed into phosphate ions under the catalysis of PPase, the Zn^2+^ triggered AIE phenomenon of Au/Ag NCs could be designed to assay the PPase activity. The Zn^2+^ triggered aggregation of Au/Ag NCs could be effectively breakdown by the combination of PPi and Zn^2+^. While PPase specifically hydrolyzed PPi to Pi, Zn^2+^ coordinated with PPi was released and recombined with Au/Ag NCs, causing the aggregation of Au/Ag NCs and the emission enhancement. With increasing the adding concentration of PPase in the mixed solution of PPi and Zn^2+^-Au/Ag NCs, the fluorescence intensity of Au/Ag NCs gradually increased (Figure 5A). With the directly adding of different concentrations of PPase into Au/Ag NCs, the fluorescence of Au/Ag NCs only exhibited a slightly change, indicating that the PPase have no obvious influence on the fluorescence of Au/Ag NCs (Supplementary Figure 9). The time-dependent fluorescence measurements showed it took 1 h to complete the reaction in the analysis system and achieve the steady of fluorescence (Supplementary Figure 10). Incubation of the Au/Ag NCs at 37°C for 1 h did not lead to much change in the emission signal (Supplementary Figure 11), demonstrating excellent stability of the Au/Ag NCs. The detection of PPase could be achieved by the relationship between the fluorescence intensity of Au/Ag NCs and the concentration of PPase (Figure 5B). The detection linear range of PPase was 1–30 U/L, according to the experimental results. The linear regression equation is y = 0.28951 x + 102.7 (R^2^ = 0.9984), where y and x are the fluorescence intensity of Au/Ag NCs and PPase concentration, respectively. The limit of detection for PPase was calculated to be 0.3 U/L. Compared with some recently reported methods for PPase activity assay based on pure metal NCs, our method has moderate cost, higher sensitivity (1.3 U/L for Cu NCs and 0.7 U/L for Ag NCs) (Tang et al., 2017; Ye et al., 2019) and better stability as Au/Ag alloy NCs typically difficult to be oxidized by the surrounding substances and process high stability in the application. Compared with analysis methods for PPi or PPase based on other fluorescent materials, our method also had higher or comparable sensitivity (Supplementary Table 2). In addition, the Cu^2+^-free, easy operation and high accuracy of our method are clear advantages.

**Figure 6:**
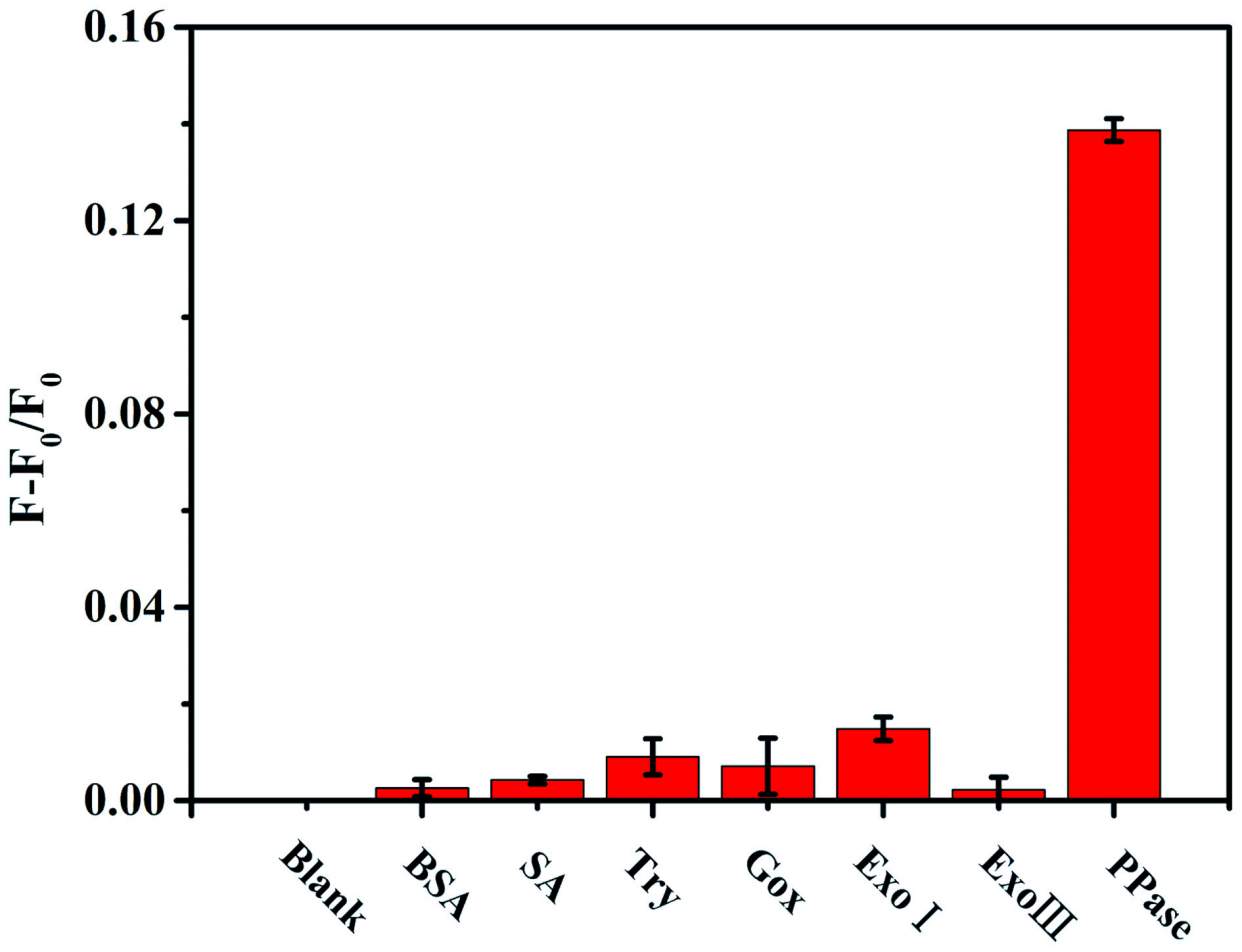
The relative fluorescence intensity of the Zn^2+^-Au/Ag NCs-PPi analysis system in the presence of different substances (F and F_0_ represents the fluorescence intensity of Au/Ag NCs in the presence and absence of the detection substances, respectively). The concentration of BSA, SA, Try, Gox is 0.1 mg/mL; Exo I, Exo III, and PPase are 30 U/L.

The response of the as-constructed analysis system to different proteins, including bovine serum albumin (BSA), streptavidin (SA), trypsin (Try), glucose oxidase (Gox), exonuclease I (Exo I), and exonuclease III (Exo III), had proved good selectivity of this method for PPase. The results of selectivity examination showed only PPase caused obvious promotion of the fluorescence of Au/Ag NCs (Figure 6), while other substances brought about little changes in fluorescence, confirming a good selectivity of the method for PPase.

To validate the practicality of this detection method for PPase, the analysis of the concentrations of PPase in human serum samples was also implemented. We spiked four concentrations of PPase (3.0, 5.0, 10.0, and 20.0 U/L) into 1% diluted human serum and investigated the recovery values of our analysis method. The obtained recovery values ranged from 96.0 to 104.0 % with a satisfactory RSD lower than 3.0%, revealing a good practicality of the proposed method for PPase detection (Table 1).

**Table 1:** Recoveries of pyrophosphatase in spiked human serum samples.

**Sample (No.)**	**Added (U/L)**	**Found (U/L)**	**Recovery (%)**	**RSD (*n* = 3)**
1	3.0	2.9	96.7	2.5 %
2	5.0	4.8	96.0	2.5 %
3	10.0	10.4	104.0	2.1 %
4	20.0	20.2	101.0	1.6 %

## Conclusion

In conclusion, we studied the AIE behaviors of water-soluble alloy Au/Ag NCs and develop a simple and sensitive strategy for the detection of PPi and PPase based on the Zn^2+^-regulated AIE of Au/Ag NCs. The alloy Au/Ag NCs exhibits unique AIE characteristics (e.g., emission shift upon aggregation) that are obviously different from pure metal NCs. Employing the AIE behaviors of this Au/Ag NCs, we constructed a simple and reliable detection method for PPi and PPase. Compared to the previous methods based on pure metal NCs, our method has the advantages of moderate cost, higher stability, and sensitivity. Moreover, the proposed method is easy to operate, low toxic, and environmentally friendly. This work not only provide a simply and new approach for the detection of PPi and PPase but also a basis for further study on the AIE properties of alloy NCs and expand their application in biological analysis.

## Data Availability Statement

The original contributions presented in the study are included in the article/Supplementary Materials, further inquiries can be directed to the corresponding author/s.

## Ethics Statement

The studies involving human participants were reviewed and approved by the ethics committee at Hubei University. Written informed consent for participation was not required for this study in accordance with the national legislation and the institutional requirements.

## Author Contributions

ZLe and ML: experimental work, data analysis, and original manuscript writing. JZ: experimental work. YX and ZLi: method design, manuscript writing, and supervision. All authors contributed to the article and approved the submitted version.

## Conflict of Interest

The authors declare that the research was conducted in the absence of any commercial or financial relationships that could be construed as a potential conflict of interest.
