# Supplementary material for: Aggregation-Induced Emission of Au/Ag Alloy Nanoclusters for Fluorescence Detection of Inorganic Pyrophosphate and Pyrophosphatase Activity
Source: Front Bioeng Biotechnol. 2021 Jan 15;8:628181. doi: 10.3389/fbioe.2020.628181 (PMC7844307; doi:10.3389/fbioe.2020.628181)
Supplement: Supplementary file 1 [file Data_Sheet_1.docx]

Supporting Information

**Aggregation-induced emission of Au/Ag alloy nanoclusters for fluorescence detection of inorganic pyrophosphate and pyrophosphatase activity**

Zhongli Lei, Jie Zhou, Miao Liang, Yan Xiao*, Zhihong Liu*

Hubei Collaborative Innovation Center for Advanced Organic Chemical Materials, Ministry of Education Key Laboratory for the Synthesis and Application of Organic Functional Molecules & College of Chemistry and Chemical Engineering, Hubei University, Wuhan 430062, P.R. China

* Corresponding author

Yan Xiao (Email: xiaoyan@hubu.edu.cn)

Zhihong Liu (Email: zhhliu@whu.edu.cn)

**Supplementary Figures and Tables**


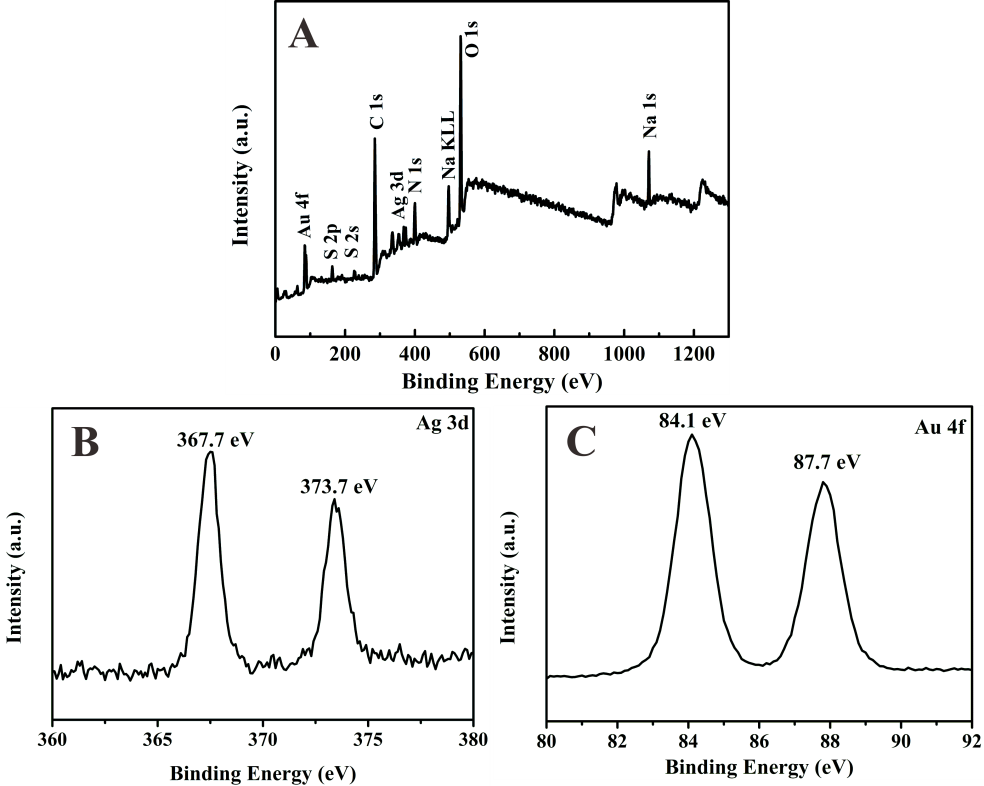


**Figure S1.** (A) X-ray photoelectron spectroscopy (XPS) survey spectrum of as-synthesized Au/Ag NCs. (B, C) Expanded spectrum in the Ag 3d (B) and Au 4f (C) region.


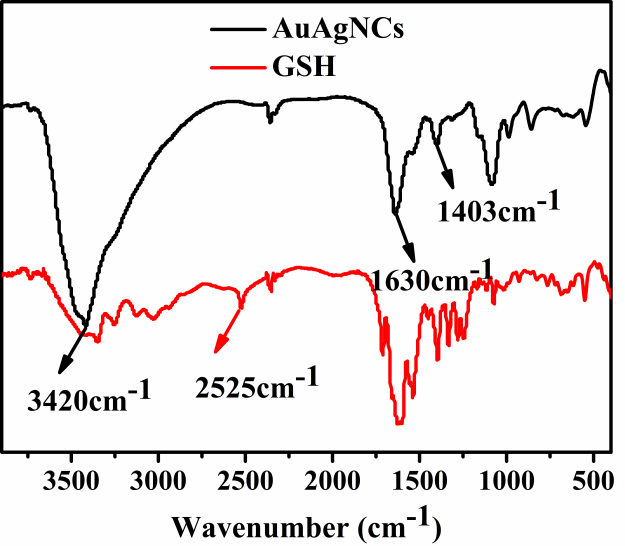


**Figure S2**. FT-IR spectra of Au/Ag NCs (black line) and GSH (red line).


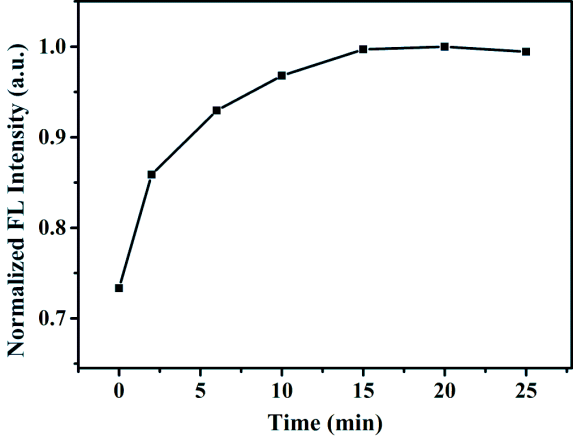


**Figure S3**. Fluorescence enhancement of 0.3 mg/mL Au/Ag NCs by 300 µM Zn^2+^ as a function of time.


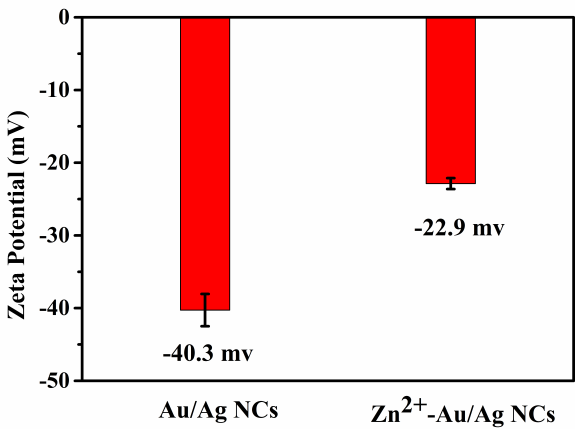


**Figure S4**. Zeta-potentials of Au/Ag NCs and Zn^2+^-Au/Ag NCs aggregates.

**
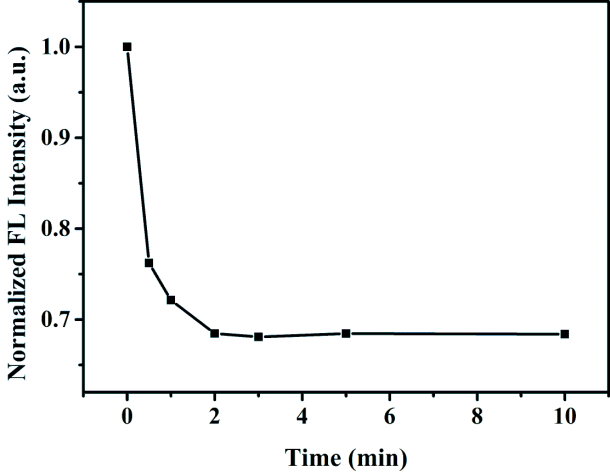
**

**Figure S5.** Fluorescence quenching of Zn^2+^-Au/Ag NCs aggregates by 400 µM PPi as a function of time.


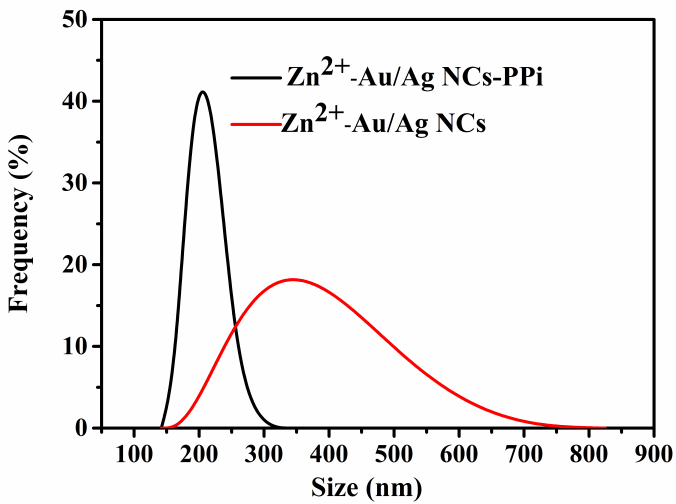


**Figure S6**. Representative dynamic light scattering (DLS) curves of Zn^2+^-Au/Ag NCs and Zn^2+^-Au/Ag NCs-PPi.

**
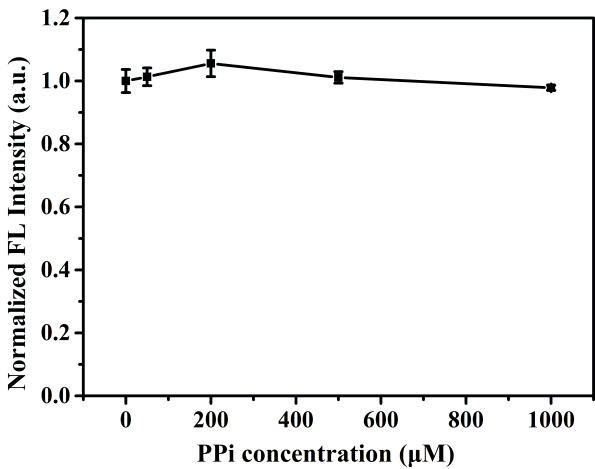
**

**Figure S7.** Normalized fluorescence intensity of Au/Ag NCs in the presence of different concentrations of PPi.


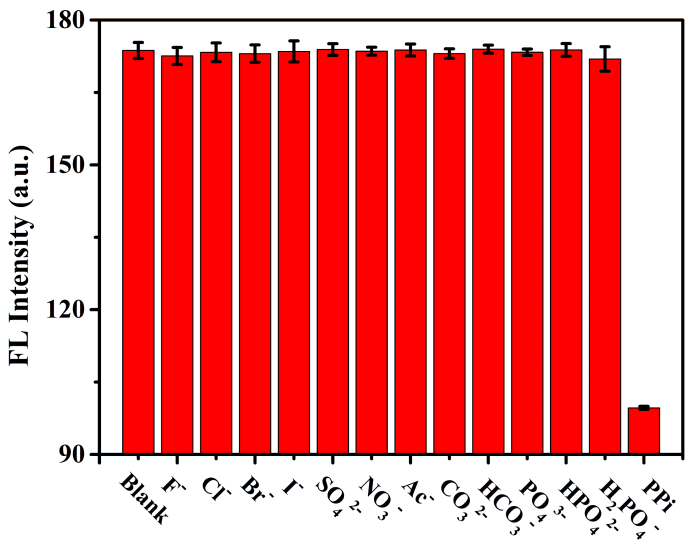


**Figure S8.** Fluorescence intensity of Zn^2+^-Au/Ag NCs aggregates in the presence of different substances. The concentrations of all the tested substances were 600 μM.


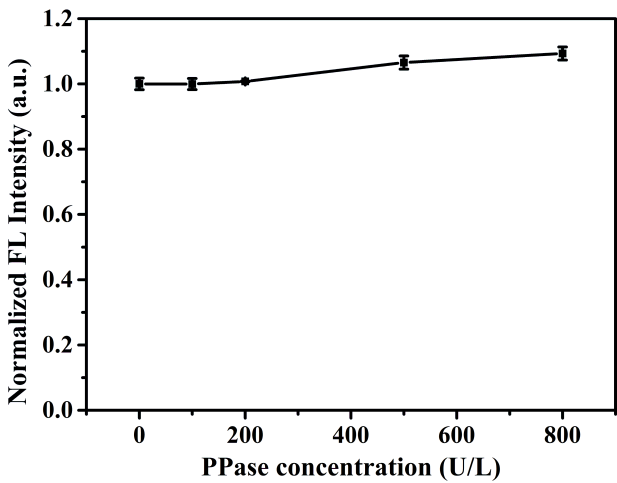


**Figure S9.** Normalized fluorescence intensity of Au/Ag NCs in the presence of concentrations of PPase.


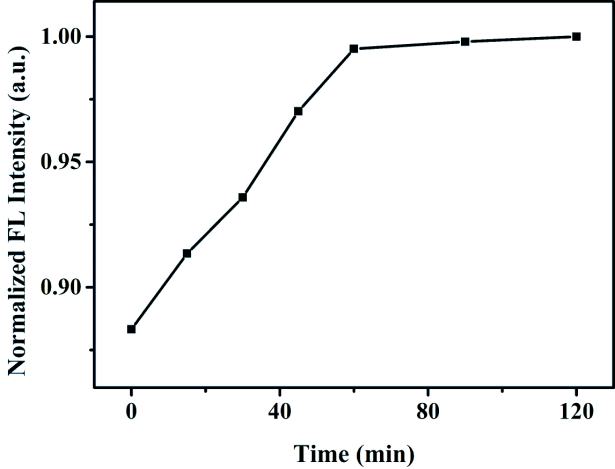


**Figure S10.** Time dependence of the fluorescence recovery of Zn^2+^-Au/Ag NCs-PPi detection system by 30 U/L PPase.


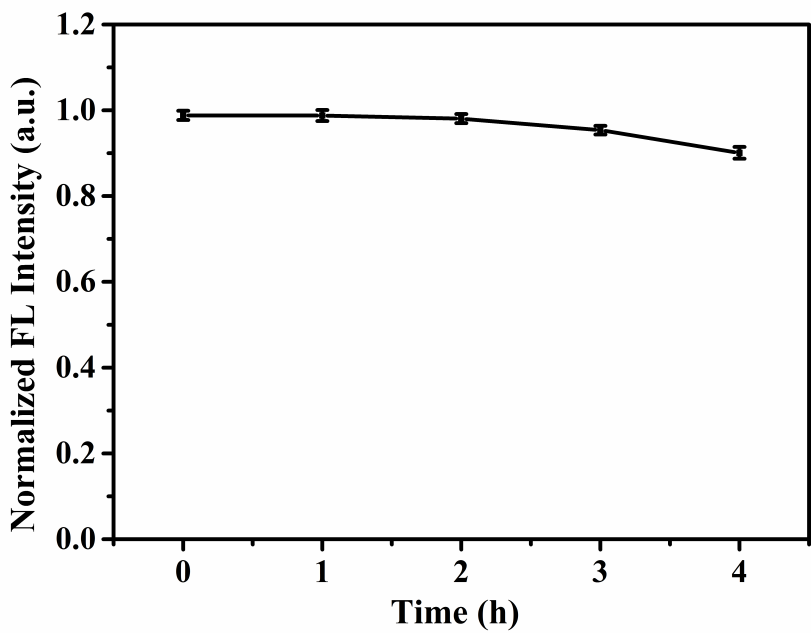


**Figure S11.** Normalized fluorescence intensity of Au/Ag NCs incubated at 37 ℃ for different time.

**Table S1.** Recovery of PPi from human serum samples.

| Sample (No.) | Added (μM) | Found (μM) | Recovery  (%) | RSD (n=3) |
| --- | --- | --- | --- | --- |
| 1 | 50.0 | 49.6 | 99.2 | 2.5 % |
| 2 | 150.0 | 148.3 | 98.9 | 0.6 % |
| 3 | 200.0 | 205.4 | 102.7 | 1.6 % |
| 4 | 300.0 | 282.1 | 94.0 | 1.3 % |

**Table S2.** Comparison of fluorometric PPi and PPase assays in analytical performance and detection route.

| Material | Involvement of Cu^2+^ | Detection limit and linear scope of PPi (μM) | Detection limit and linear scope of PPase (U/L) | References |
| --- | --- | --- | --- | --- |
| 3-azidocoumarins | Yes | -  - | 0.2  0.5-10 | (Xu et al., 2015) |
| o-Phenylenediamine | Yes | 2.0  2.0-20 | 0.2  0.2-50 | (Sun et al., 2016) |
| Graphene quantum dots | Yes | -  - | 1.0  1.0-200 | (Zhu et al., 2016) |
| Au NCs | Yes | 1.0  1.0-20 | 0.2  1.0-20.0 | (Sun et al., 2014) |
| Ag NCs | No | 0.7  3.8-110.0 | 0.7  2.1-35 | (Tang et al., 2017) |
| Cu NCs | No | -  - | 1.3  3-40 | (Ye et al., 2019) |
| Au/Ag NCs | No | 3.2  10-400 | 0.3  1-30 | This work |

**REFERENCES**

Sun, J., Wang, B., Zhao, X., Li, Z. J. and Yang, X. (2016). Fluorescent and colorimetric dual-readout assay for inorganic pyrophosphatase with Cu^2+^-triggered oxidation of o-phenylenediamine. Anal. Chem. 88, 1355-1361. doi:10.1021/acs.analchem.5b03848

Sun, J., Yang, F., Zhao, D. and Yang, X. (2014). Highly sensitive real-time assay of inorganic pyrophosphatase activity based on the fluorescent gold nanoclusters. Anal Chem. 86, 7883-7889. doi:10.1021/ac501814u

Tang, C., Feng, H., Huang, Y. and Qian, Z. (2017). Reversible luminescent nanoswitches based on aggregation-induced emission enhancement of silver nanoclusters for luminescence turn-on assay of inorganic pyrophosphatase activity. Anal. Chem. 89, 4994-5002. doi:10.1021/acs.analchem.7b00319

Xu, K., Chen, Z., Zhou, L., Zheng, O., Wu, X., Guo, L., Qiu, B., Lin, Z. and Chen, G. (2015). Fluorometric method for inorganic pyrophosphatase activity detection and inhibitor screening based on click chemistry. Anal. Chem. 87, 816-820. doi:10.1021/ac503958r

Ye, M., Yu, Y., Lin, B., Cai, Y., Cao, Y., Guo, M. and Zhu, D. (2019). Copper nanoclusters reversible switches based on ions-triggered for detection of inorganic pyrophosphatase activity. Sens. Actuators, B. 284, 36-44. doi:10.1016/j.snb.2018.12.092

Zhu, X., Liu, J., Peng, H., Jiang, J. and Yu, R. (2016). A novel fluorescence assay for inorganic pyrophosphatase based on modulated aggregation of graphene quantum dots. Analyst. 141, 251-255. doi:10.1039/c5an01937k
